# The effects of DOPO modified Co-based metalorganic framework on flame retardancy, stiffness and thermal stability of epoxy resin

**DOI:** 10.1039/d0ra08982f

**Published:** 2021-02-10

**Authors:** Liyun Cai, Fei Xin, Congcong Zhai, Yu Chen, Bo Xu, Xiangmei Li

**Affiliations:** College of Chemistry and Materials Engineering, Beijing Technology and Business University Beijing 100048 People's Republic of China clydeyx@163.com xinfei@th.btbu.edu.cn 15501099906@163.com xubo@btbu.edu.cn +86-10-68985531; Beijing Huateng Hightech Co., Ltd. Beijing 100048 People's Republic of China chenyu6911@163.com; School of Materials Science & Engneering, Beijing Institute of Technology Beijing 100081 People's Republic of China bjlglxm@bit.edu.cn +86-10-68913066

## Abstract

In this work, the effect of a modified metal organic framework material on the fire resistance and mechanical properties of epoxy resin (EP) has been explored. The cobalt based metal organic framework (ZIF-67) was synthesized from an organic ligand with a Schiff base structure. Then DOPO@ZIF-67 was synthesized by modifying ZIF-67 with 9,10-dihydro-9-oxa-10-phosphaphenanthrene-10-oxide (DOPO), and its effect on EP modification was explored. Compared with the pure EP, 4% DOPO@ZIF-67/EP passed the UL94 V-0 level and the ultimate oxygen index (LOI) reached 32.1%. The SEM pictures of carbon residue indicated that DOPO@ZIF-67 formed a more continuous and dense microstructure, which can enhance the thermal barrier and the physical barrier effect. The addition of DOPO@ZIF-67 also can effectively improve the stiffness and damping coefficient of EP composite material. The porous skeleton structure of DOPO@ZIF-67 can endow EP with rigidity and flame-retardant properties. Furthermore, the existence of DOPO made the combination of ZIF-67 with EP better. The results of this study suggest that DOPO@ZIF-67 may be a good additive for modification of the properties of epoxy thermosetting materials.

## Abstract 2

The DOPO@ZIF-67 promoted the carbonization process of the EP flame retardant composites, and the rigidity and damping coefficient of EP composites are also improved compared with pure EP.

## Graphical abstract

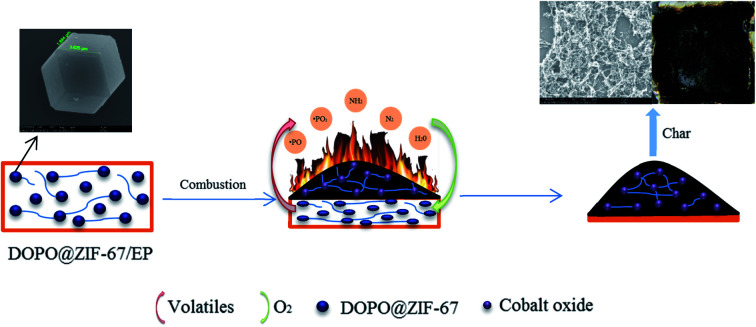

## 1. Introduction

Due to the requirements for materials performance and the enhancement of standards, plastic materials are widely used due to their excellent properties. However, with frequent fires and serious losses to society led by the widespread use of the flammable and combustible plastic materials, the flame-retardant modification of plastics, various flame-retardant modified materials and flame retardants have attracted considerable attention.

Metal–organic frameworks (MOFs) are coordinated polymers with ordered three-dimensional nanostructures,^1^ which are composed of metal ions and organic ligands with moderately strong coordination.^2^ In the synthesis of MOFs, the presence of solvents provides the necessary fluidity for metal ions and organic ligands to complete the required coordination reaction. In the absence of solvents, coordination polymerization can be triggered by ball-milling, extrusion and heating.^3^ Metal–organic frameworks are mainly used in adsorption,^4^ purification of water and air,^5^ electrochemistry and other application.

Different MOFs materials have been synthesized by different applications. For example, materials derived from ZIFs such as metal–organic framework-derived N-doped carbon nanotubes (MOF-NCNTs),^6^ bimetallic zinc/cobalt zeolitic imidazolate frameworks nanoplate arrays (Zn/Co-ZIF NPAs),^7^ bicomponent metal organic framework based on nanofibers (Ag-MOFs@CNF@ZIF-8),^8^ magnetic metal–organic framework nanocomposite (ZIF-8@SiO_2_@MnFe_2_O_4_),^9^ zeolitic imidazolate frameworks-8 loaded onto the surface of graphene (ZIF-8/RGO),^10^ novel honeycomb-like mesoporous aluminum hydroxide (pATH)^11^ and so on. Materials derived from MILs materials such as amino-functionalized Cr-based metal–organic frameworks (Pd/MIL 101-NH_2_),^12^ iron-based metal organic framework (NH_2_–Fe-MIL-88B)^13^ and so on. Materials derived from BTC materials such as new type of mesoporous carbon nanofibers (OMCN@NiCo_2_O_4_),^14^ Cu/Fe-BTC,^15^ nitrogen-containing microporous carbon composite copper-based metal organic framework (NC–Cu-BTC)^16^ and so on. Materials derived from UiO materials such as UiO-66 modified with thiol groups (UiO-66-SH),^17^ metal–organic frameworks@cellulose aerogels composite materials (UiO-66@CA)^18^ and so on. Materials derived from Co-MOFs materials such as nitrogen-doped carbon plate composites modified by cobalt-based metal organic framework (Co_3_O_4_/NC),^19^ Ni-doped Co-based metal organic framework (Ni/Co-MOF),^20^ layered Co-based metal–organic framework,^21^ Co-based metal–organic framework with phosphorus-containing structure (P-MOF)^22^ and so on. As is known to all, the metal organic framework can form various materials with different properties by reacting with other chemicals. At the same time, it can be seen from the structures of classical types of MOFs and the various derived MOFs materials that flame retardant elements, such as N and P, are contained in their skeleton structures. Therefore, we prepare an excellent flame-retardant materials based on MOFs material.

In recent years, scholars have gradually begun to try to apply MOFs to the flame retardant modification of resin matrix. Wenzong Xu *et al.*,^23^ prepared functionalized reduced graphene oxide (RGO) with Co-ZIF (zeolitic imidazolate frameworks-67) adsorbed borate ions (ZIF-67/RGO-B) which was used to reduce the fire risk of epoxy resin (EP). Hailin Guo *et al.*,^24^ synthesized a kind of flame-retardant CoAl-LDH@ZIF-67 hybrids which was constructed by *in situ* growth of nanometer scale ZIF-67 crystallites on CoAl-LDH nanoplates to improve the flame-retardant properties of epoxy resin. Yanbei Hou *et al.*,^25^ synthesized ZIFs and applied it to flame retardant modification of PS resin materials.

EP has been widely used because of its excellent electrical insulation and corrosion resistance and other excellent properties.^26^ However, the flammability of EP limits the application in many ways. In order to expand its application range, the flame retardancy of EP was improved by adding various flame retardants.^27^

As phosphorus flame retardants have the characteristics of environmental protection, high efficiency and non-toxicity, there are many kinds of phosphorus flame retardants had been applicated, making them the most likely alternative to halogen flame retardants to be widely used flame retardant products. 9,10-dihydro-9-oxa-10-phosphaphenanthrene-10-oxide (DOPO) shows remarkable flame retardant effect in epoxy resin (EP).^27^

In this work, DOPO was used to react with ZIF-67 by the –C=N– structure, and the product was applied in EP to study the effect on the flame retardant and mechanical properties of EP. The flame retardant behavior of EP composites was investigated *via* the limiting oxygen index (LOI), the vertical burning (UL 94), cone calorimeter test (CONE) and so on. As for the mechanical properties of EP composites, were mainly tested by dynamic mechanical analysis (DMA) and unnotched Charpy impact test.

## 2. Materials and methods

### 2.1 Materials

2-Methylimidazole (2MI) (≥99%), cobaltous nitrate hexahydrate (Co(NO_3_)_2_·6H_2_O) (≥99%) and tetrahydrofuran (THF) (≥99.5%) were obtained from Beijing Bailingwei Technology Co., Ltd., China. Methanol (AR) and ethanol (GR) were purchased from Beijing Chemical Plant. 9,10-Dihydro-9-oxa-10-phosphaphenanthrene-10-oxide (DOPO) was provided by Jiangyin Hanfeng Chemical Co., Ltd., China. The epoxy resin was purchased from Nantong Star Synthetic Materials Co., Ltd., China. 4,4′-Diamino-diphenyl methane (DDM) was purchased from Macklin Biochemical Technology Co., Ltd., Shanghai of China.

### 2.2 Synthesis of the ZIF-67

**Fig. 1:**
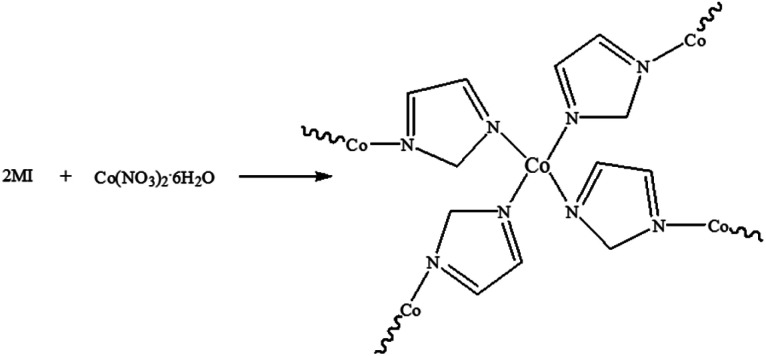
Synthesis of ZIF-67.

The synthesis of ZIF-67 is based on typical synthesis methods.^28^ The synthetic route of ZIF-67 is shown in Fig. 1. 2-Methylimidazole and cobalt nitrate hexahydrate were added into methanol/ethanol (v/v = 1 : 1) mixed solution respectively, stirring to make the solution uniform, then mixing the two solution and stirring for several seconds to make the mixed solution uniform. The mixed solution was then aged for 20 h. The precipitation was filtered and washed with ethanol, and the filtered product was dried in the oven at 100 °C for 5 h.

### 2.3 Synthesis of the DOPO@ZIF-67

**Fig. 2:**
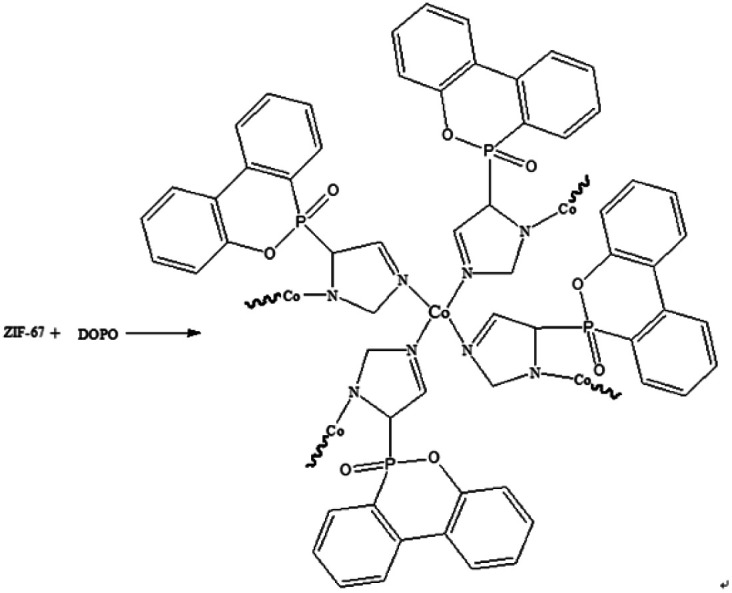
Synthesis of DOPO@ZIF-67.

ZIF-67 (0.116 g) was dispersed in ethanol (40 ml) and label it as A. DOPO (0.216 g) was dissolved in ethanol (20 ml) and label it as B. Pour solution B into solution A and stir at room temperature for 30 min to evenly disperse. The reaction temperature was then raised to 50 °C for 5 h. After 5 h, the blue powder product was obtained by distillation at reduced pressure, and washed several times using water/ethanol (v/v = 1 : 1) mixture. Finally, the product was dried in a blast oven at 100 °C for 5 h. The yield was 68.7%. The synthetic route of DOPO@ZIF-67 is shown in Fig. 2.

### 2.4 Preparation of EP composites

DOPO@ZIF-67 were added to epoxy resin, stirred at 120 °C for 1 h, DDM was added to mix evenly, then pumped into vacuum oven for 3 minutes, cured at 120 °C for 2 h, 170 °C for 4 h. Then the DOPO@ZIF-67/EP splines with 1%, 2%, 3% and 4% DOPO@ZIF-67 addition can be obtained by demoulding.

### 2.5 Characterizations

Fourier transform infrared spectroscopy (FTIR) was using Nicolet i N10MX spectrometer. The powdered samples were thoroughly mixed with KBr and then pressed into pellets.

X-ray diffraction (XRD) was tested with XPert Powder (PANalytical, Netherlands) and the diffraction angle was from 10° to 40°.

The morphology was characterized using a Quanta 250 (FEI, USA) scanning electron microscope (SEM) at an acceleration voltage of 15 kV.

The composition of the materials synthesized was determined by X-Ray Fluorescence (XRF) (XRF-1800, Shimadzu). The conditions of analysis were as follows: vacuum atmosphere, using 40 kV scans, which determine the regions of the elements P, O, N, Co, C and H.

Thermogravimetric analysis (TGA) was conducted on a TA Q5000IR with a heating rate of 20 °C min^−1^ under N_2_ atmosphere from 50 to 700 °C.

LOI values were measured on a FTT (Fire Testing Technology, UK) according to ASTM D2863-97, and dimensions of samples were 130.0 mm × 6.5 mm × 3.0 mm.

UL 94 combustion level was using an FTT0082 instrument according to ASTM D 3801 (sample dimensions: 125 mm × 12.7 mm × 3.2 mm).

The fire behavior was characterized by a FTT0007 (Fire Testing Technology, UK) cone calorimeter (CONE) according to ISO 5660 (sample size: 100.0 mm × 100.0 mm × 3.0 mm) under an external heat flux of 50 kW m^−2^.

The morphology of residual chars after CONE test was characterized using a Quanta 250 (FEI, USA) scanning electron microscope (SEM) at an acceleration voltage of 15 kV.

The dynamic mechanical analysis (DMA) was performed using a DMA Instrument DMA242C (NETZSCH, Germany). Tests were run under controlled amplitude in the dual cantilever mode, with an oscillation frequency of 1.0 Hz. The heating rate of 2 °C min^−1^ was ramped from 30 °C to 220 °C, under controlled sinusoidal strain.

The impact strength of epoxy composites was evaluated on a Zwick HIT50P impact test machine according to ISO 13802 with a 2 J pendulum. The results were the average for five times. The dimensions of the specimens were 80 mm × 10 mm × 4 mm.

## 3. Results and discussion

### 3.1 Characterization of ZIF-67

**Fig. 3:**
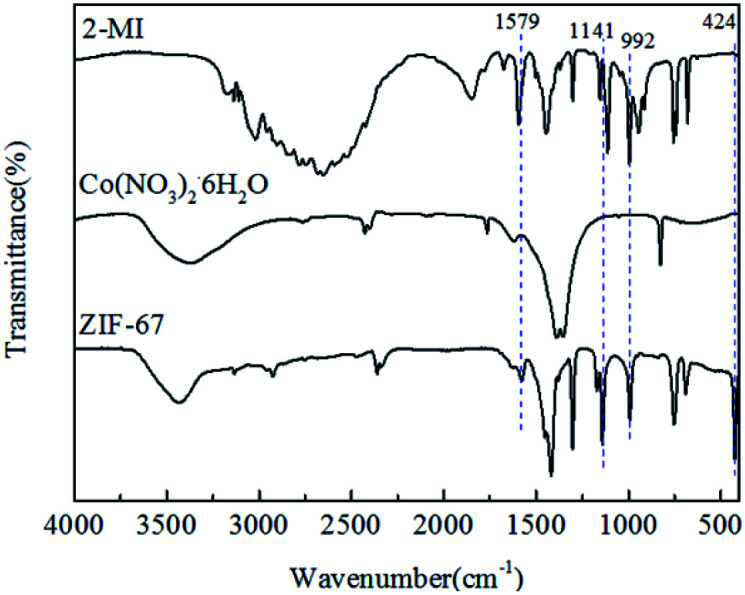
FTIR spectra of 2MI, Co(NO_3_)_2_·6H_2_O and ZIF-67.

**Fig. 4:**
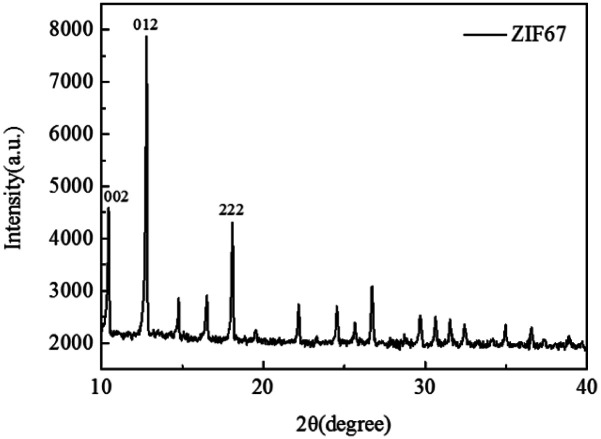
XRD spectra of ZIF-67.

The FTIR patterns of synthetic products and raw materials are shown in Fig. 3. A clear indication of ZIF-67 peaks is primarily attributed to the ligand 2-methylimidazole. The peak at 1579 cm^−1^ is due to the C=N stretching vibration of imidazole ligands, 1141 cm^−1^ is assigned to non-planar vibration band of imidazole ring, 992 cm^−1^ is attributed to the planar bending vibration band of imidazole ring and 424 cm^−1^ is correspond to the Co–N.^23^ The corresponding characteristic bands appear in the infrared spectrogram of synthesized products, which preliminarily indicates that the synthesized product is ZIF-67. The crystal morphology of ZIF-67 is characterized by XRD. The XRD spectrum of ZIF-67 is shown in Fig. 4. The representative reflections of ZIF-67, (002), (012) and (222) reflections, correspond to standard ZIF-67 structure.^22,23^ The result of FTIR and XRD indicated that the synthesis of ZIF-67 was successful.

**Fig. 5:**
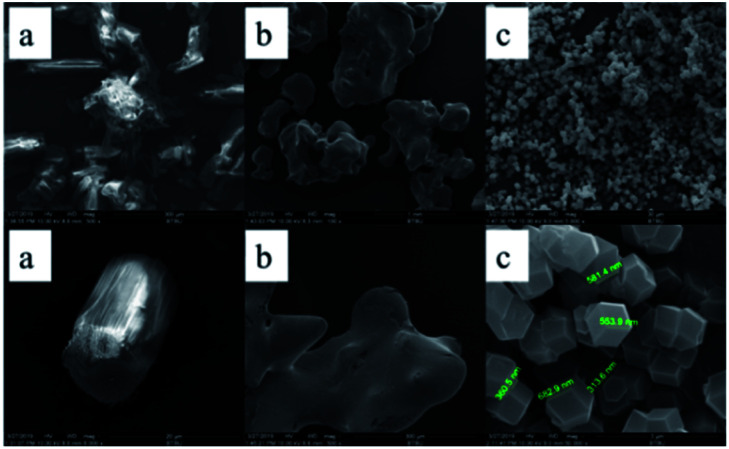
SEM images of 2MI, Co(NO_3_)_2_·6H_2_O and ZIF-67: (a) 2MI; (b) Co(NO_3_)_2_·6H_2_O; (c) ZIF-67.

The SEM images are applied to demonstrate the microstructures of the ZIF-67. From the Fig. 5, it can be clearly obtained that most of the synthesized products have very regular polyhedral structure and the size of ZIF-67 is between 300–800 nm, which is the same as the description of ZIF-67 synthesized by a typical synthesis method.^28^ The morphology of 2-methylimidazole and cobalt nitrate hexahydrate is irregularly, the different morphologies between the synthesized products and the raw materials strongly prove the successful synthesis of ZIF-67.

### 3.2 Characterization of DOPO@ZIF-67

**Fig. 6:**
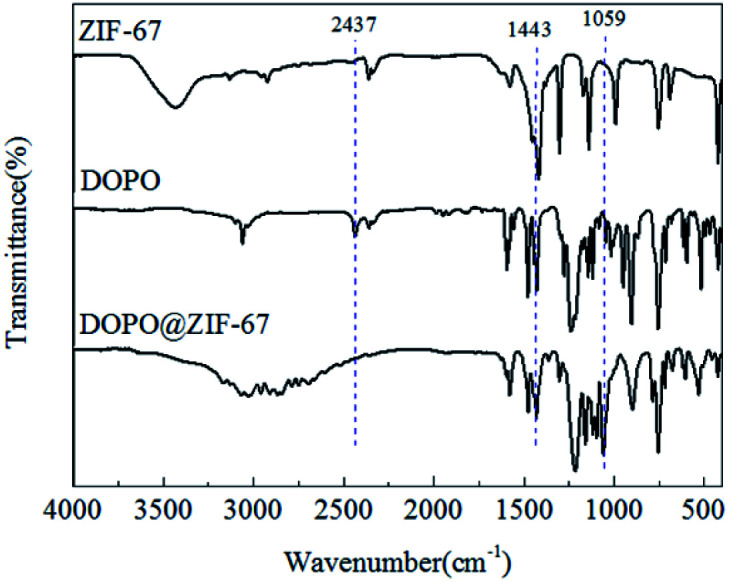
FTIR spectra of ZIF-67, DOPO and DOPO@ZIF-67.

Theoretically, the P–H functional group in DOPO will react with the –C=N– double bond on ZIF-67 to form a coating structure on the surface of ZIF-67. Fig. 6 depicts the infrared spectra of ZIF-67, DOPO and DOPO@ZIF-67. The peak of DOPO at 2437 and 1443 cm^−1^ are due to the P–H and P–Ph. Since the P–Ph bond exists in the chemical structure before and after the reaction, the infrared characteristic peak at 1443 cm^−1^ still exists, but the peak at 2437 cm^−1^ which belong to P–H bond disappears. As showed in the infrared spectra of DOPO@ZIF-67, a new peak appeared at 1059 cm^−1^, ascribed to the vibration of newly formed C–P bond.^21^ The disappearance of P–H bond and the appearance of new characteristic peaks can confirm the formation of DOPO@ZIF-67.

**Fig. 7:**
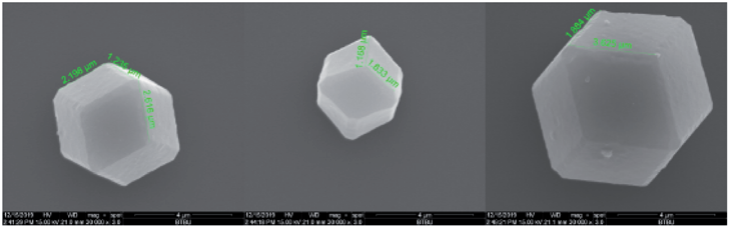
SEM images of DOPO@ZIF-67.

ZIF-67 which appears as a purple powder reacts with DOPO to produce a blue powder product, which visually demonstrates the reaction. To investigate the effect of DOPO on ZIF-67 microstructure, SEM images were provided. As shown in Fig. 7, the smooth surface of ZIF (as shown in Fig. 4c) is replaced by a rough appearance, while the particle size is significantly increased, which can be attributed to the DOPO coating.

XRF test results are shown in Table 1. The results showed that the proportion of phosphorus (P) in DOPO@ZIF-67 is 5.8570%, then the covering rate of DOPO in DOPO@ZIF-67 can be calculate as 40.57%, because in molecular structure, there are only DOPO contains P element.

**Table 1:** Percentage of the main chemical elements of DOPO@ZIF-67

Samples	Element	Percentage (%)
DOPO@ZIF-67	N	16.42
	O	7.67
	Co	7.66
	P	5.85
	CH	62.40

**Fig. 8:**
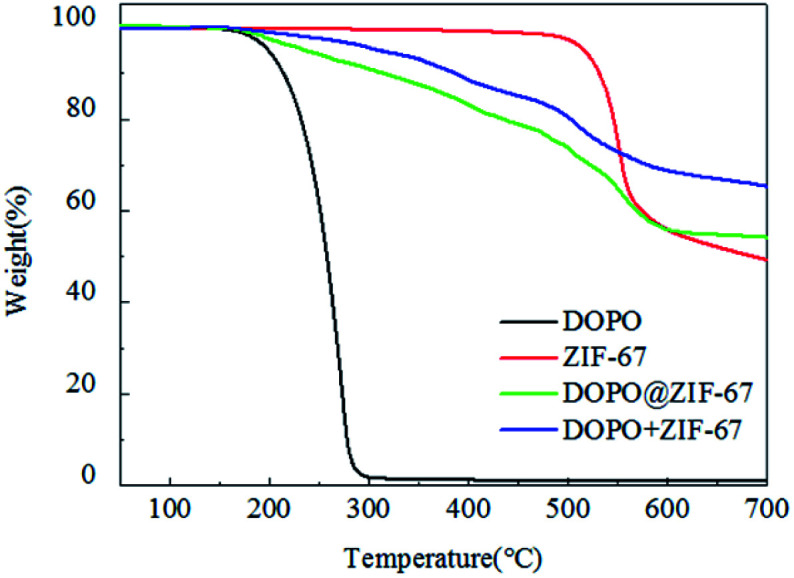
TGA curves of ZIF-67, DOPO, DOPO@ZIF-67 and DOPO + ZIF-67.

Thermal stability of flame retardant is showed in Fig. 8 (specific values can be obtained from Table 2). Among them, *T*_d,5%_ represents the temperature at which the mass loss of the sample reaches 5% during decomposition. When the mass loss reaches 5%, the decomposition temperature of DOPO@ZIF-67 is between ZIF-67 and DOPO. However, according to the mass of char yield, the char yield of DOPO@ZIF-67 is larger than ZIF-67 and DOPO. According to the TG curve variation trend, the thermal stability of DOPO@ZIF-67 is improved, and the decomposition curve is smooth decreased. DOPO is coated on ZIF-67, so the *T*_d,5%_ of DOPO@ZIF-67 was close to that of DOPO. The slope of the curve is lower than that of DOPO in the decomposition stage, indicating that the combination of ZIF-67 and DOPO can significantly improve the thermal stability of DOPO. The mixture of DOPO and ZIF-67 with the same ration as DOPO@ZIF-67 is named DOPO + ZIF-67. The TG curve of the blends is different from the curve of DOPO@ZIF-67. The early decomposition of DOPO@ZIF-67 facilitates the formation of metal oxides to facilitate the carbonization process during the combustion of the composite, thus the epoxy matrix can be protected better. It indicates that ZIF-67 has reacted with DOPO and the thermal stability is improved.

**Table 2:** Parts data of TGA curves of ZIF-67, DOPO and DOPO@ZIF-67

Samples	(*T*_d,5%_)/°C	Residue at 700 °C/%
ZIF-67	516	49.23
DOPO	198	1.14
DOPO@ZIF-67	234	54.21
DOPO + ZIF-67	310	65.29

### 3.3 Thermal properties of DOPO@ZIF-67/EP systems

**Fig. 9:**
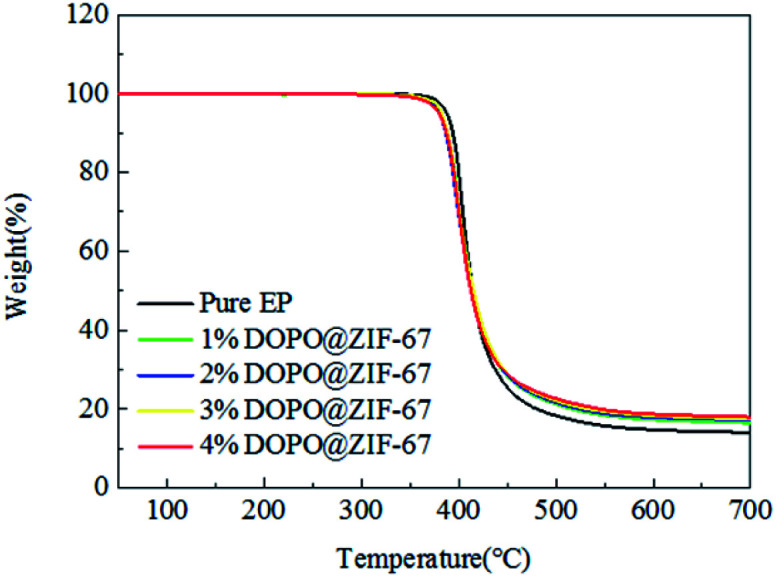
TGA curves of pure EP and DOPO@ZIF-67/EP.

Thermal stability of polymer materials is one of the most important properties in its application. Fig. 9 shows TG curves of EP flame retardant composites (specific values can be obtained from Table 3). From the data of Table 3, *T*_d,5%_ of pure EP is 387 °C. As for the DOPO@ZIF-67/EP system, the *T*_d,5%_ of the flame retardant composites are decreased a bit. The *T*_d,5%_ of EP flame retardant system decrease due to the catalytic degradation and thermal conductivity of flame retardants. According to Table 3, the mass of pure EP char yield is only 13.99% at 700 °C, however, the char yield of DOPO@ZIF-67/EP system are obviously higher than that of pure EP. It indicates that DOPO@ZIF-67 has a better effect on promoting carbonization. High carbon yield helps to reduce the further degradation of flame retardant composites, and reduce oxygen exchange and heat and mass transfer. It can be concluded that the addition of flame retardant improves the thermal stability of the composites and contributes to the formation of char.

**Table 3:** Parts data of TGA curves of pure EP and DOPO@ZIF-67/EP

Samples	(*T*_d,5%_)/°C	Residue at 700 °C/%
Pure EP	387	13.99
1% DOPO@ZIF-67	381	16.51
2% DOPO@ZIF-67	379	17.05
3% DOPO@ZIF-67	382	17.39
4% DOPO@ZIF-67	379	17.95

### 3.4 Flame retardancy of epoxy composites

The LOI and UL94 tests are two methods to evaluate the combustion performance of composites. The LOI values and UL94 test data of flame retardant EP were shown in Table 4. The LOI values of flame retardant composites increase with the increase of flame retardant dosage. The LOI of pure EP is 25.3%, while that of 4% DOPO@ZIF-67/EP was 32.1%. According to the UL94 test results, with the content of the flame retardant increases, when the addition of flame retardant is 4%, the flame retardant EP material passes the V-0 test condition. Compared with the 4% DOPO@ZIF-67/EP, The LOI of 4% DOPO + ZIF-67/EP was only 28.8% and passed UL 94 V-2 test. The results showed that the flame retardant effect of DOPO@ZIF-67 is better than that of the mixture of DOPO and ZIF-67. The reason for this result is that DOPO@ZIF-67 achieves the intermolecular coeffect between DOPO and ZIF-67 through chemical modification, which makes its flame retardant effect for EP better. Combined with TG results, the addition of DOPO@ZIF-67 promoted the carbon formation process of polymer matrix in the combustion process, and also played a role in improving the LOI and UL94 levels of epoxy resin. Therefore, DOPO@ZIF-67 has a good effect on flame retardant modification of EP.

**Table 4:** Vertical combustion and LOI test results of flame-retardant EP

Sample	LOI	UL 94	
		Droplet	Level
Pure EP	25.3	Yes	NR
1% DOPO@ZIF-67	28.5	No	V-2
2% DOPO@ZIF-67	29.7	No	V-2
3% DOPO@ZIF-67	30.9	No	V-1
4% DOPO@ZIF-67	32.1	No	V-0
4% DOPO + ZIF-67	28.8.	No	V-2

The cone calorimeter (CONE) test can assess the fire risk of the flame retardant modified polymers because it can simulate a real fire scenario. In the CONE test, the heat flux is 50 kW m^−1^.^2^ The combustion performance of pure EP, 1% DOPO@ZIF-67/EP and 4% DOPO@ZIF-67/EP are characterized by some main parameters: the peak heat release rate (pHRR), total heat release rate (THR), the total smoke release (TSR), the total mass loss (TML), the time to ignition (TTI), average effective combustion heat (av-EHC), average carbon dioxide production (av-CO_2_) and average carbon monoxide production (av-CO) and so on.

**Fig. 10:**
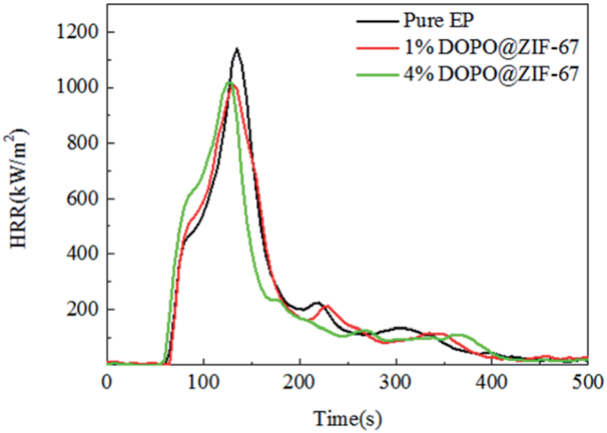
HRR curves of pure EP, 1% DOPO@ZIF-67/EP and 4% DOPO@ZIF-67/EP.

**Fig. 11:**
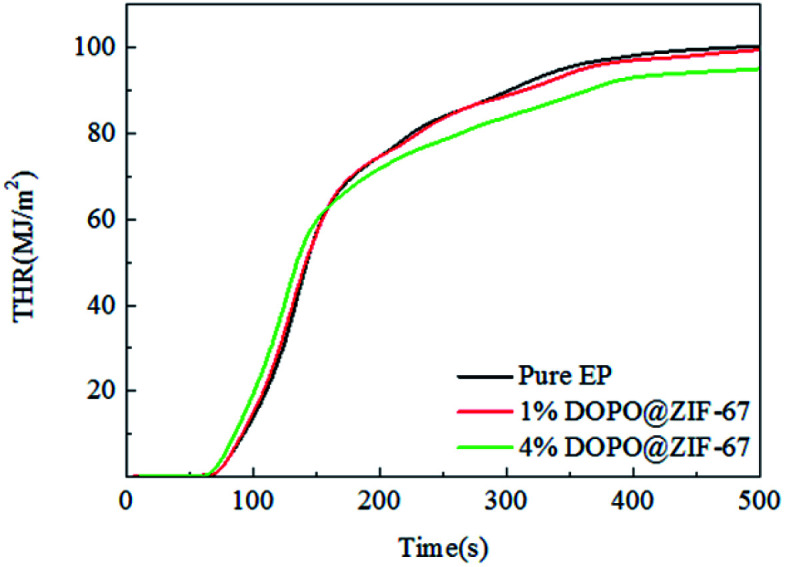
THR curves of pure EP, 1% DOPO@ZIF-67/EP and 4% DOPO@ZIF-67/EP.

As can be seen from the HRR curves of pure EP, 1% DOPO@ZIF-67/EP and 4% DOPO@ZIF-67/EP in Fig. 10, the occurrence time of pHRR in the flame retardant EP composites are earlier than the pure sample, indicating that the flame retardant released heat before the burning of the resin material, thus protecting the resin substrate. However, at around 350 s, the pure EP sample was completely burned and no heat was released. The small peak values of the composites added with flame retardant are supposed to be the heat released due to the blow out effect of DOPO. The values of pHRR of flame-retardant EP composites are decreased compared with that of pure EP, but the reason why the value of pHRR of flame-retardant composites with different additive amounts of flame retardant was almost the same is that the flame retardant promotes the formation of carbon layer, and the dense carbon layer hinders the release of heat. Fig. 11 shows the THR curves of pure EP, 1% DOPO@ZIF-67/EP and 4% DOPO@ZIF-67/EP. As can be seen from the THR curves, with the increase of flame retardant content, the THR value of flame-retardant composites decreased more significantly. According to the data shown in Table 3, the THR of pure EP is 100 MJ m^−2^, and 1% DOPO@ZIF-67/EP is 99 MJ m^−2^, while that of 4% DOPO@ZIF-67/EP is 94 MJ m^−2^. The main reason for the decrease of the pHRR and THR of the flame retardant composites is that the CO_3_O_4_ produced during combustion of ZIF-67 contributes to the formation of carbon layer,^23^ thus inhibiting the release of combustion gases and heat transfer during decomposition of the composites.

**Fig. 12:**
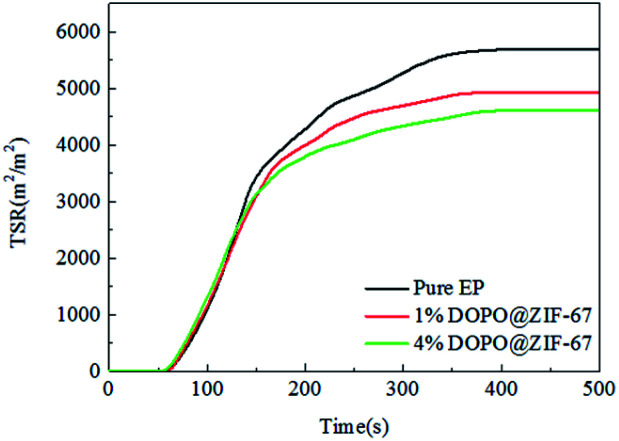
TSR curves of pure EP, 1% DOPO@ZIF-67/EP and 4% DOPO@ZIF-67/EP.

The TSR curves of EP composites during the combustion process are shown in Fig. 12. According to the curves from Fig. 12, compared with the pure EP, the TSR of the flame retardant composites decrease significantly, and with the increase of the amount of flame retardant, the value of TSR further decrease. This may be due to the fact that the flame retardant help to promote the carbon formation reaction in the combustion process of flame retardant composites, which is conducive to the formation of dense carbon layer, thus reducing the smoke emission and having the effect of smoke suppression.

As shown in Table 5, it is the partial cone calorimeter test data of flame retardant composites. The results show that the charring process of flame retardant composites were promoted in the combustion process, and a dense charring layer was generated on the surface of the matrix, which played a role in isolating oxygen and heat.^29^ According to the above results, it shows that DOPO@ZIF-67 could promote the carbon formation of EP matrix.

**Table 5:** Partial cone calorimeter test data of flame-retardant EP

Samples	TTI (s)	av-EHC (MJ kg^−1^)	pHRR (kW m^−2^)	THR (MJ m^−2^)	*W* (CCR) (%)	TML (g)	av-CO_2_ (kg kg^−1^)	av-CO (kg kg^−1^)
EP	56	23.5	1140	100	8.21	37.72	1.91	0.08
1% DOPO@ZIF-67	60	23.6	1014	99	11.69	36.56	1.90	0.09
4% DOPO@ZIF-67	53	23.8	1016	94	12.53	35.34	1.92	0.09

### 3.5 Morphology of residual chars

**Fig. 13:**
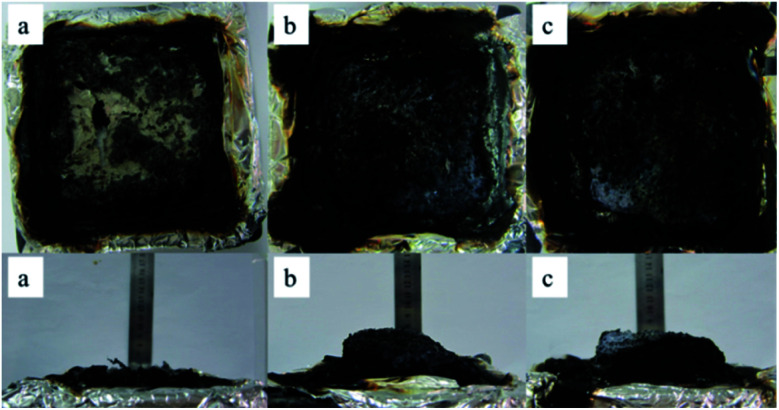
The macroscopic morphology of char residues: (a) pure EP; (b) 1% DOPO@ZIF-67/EP; (c) 4% DOPO@ZIF-67/EP.

**Fig. 14:**
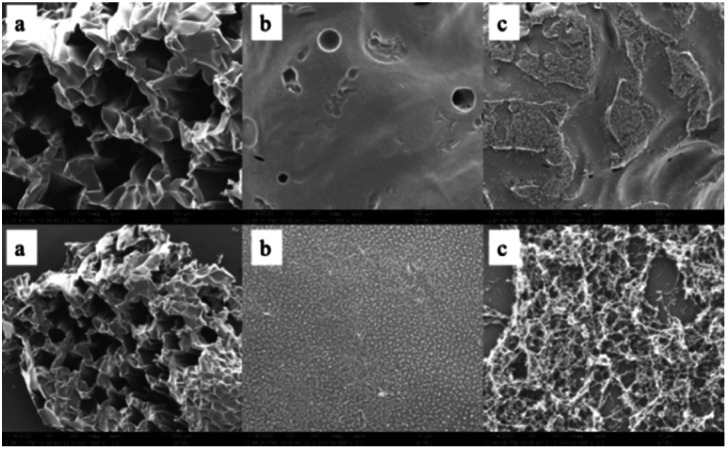
SEM images of pure EP, 1% DOPO@ZIF-67/EP and 4% DOPO@ZIF-67/EP: (a) pure EP; (b) 1% DOPO@ZIF-67/EP; (c) 4% DOPO@ZIF-67/EP.

The macro-morphology of carbon residue after CONE test can be obtained from Fig. 13, Fig. 14 shows the microstructure of outer carbon residue obtained by SEM test. As can be seen from Fig. 13, the char residual of pure EP is significantly less than 1% DOPO@ZIF-67/EP and 4% DOPO@ZIF-67/EP, while 4% DOPO@ZIF-67/EP showed more visible carbonized layers. In the SEM images of the char residues, the surface of flame retardant composites shows more continuous and dense structure, which helps to form a barrier to insulate heat and oxygen. Fig. 14 shows that there are a large number of small particles on the surface of 1% DOPO@ZIF-67/EP, while the surface of 4% DOPO@ZIF-67/EP covers the network structure, which means that the increase of the amount of flame retardant is helpful to promoted the formation of the network structure in the combustion process of the flame retardant composites. The more obvious network structure is more conducive to the formation of a dense surface, showing a better physical barrier effect, so as to protect the underlying substrate.^24^ It can be concluded that DOPO@ZIF-67 is conducive to promoting the carbonization process of EP during the combustion process.

### 3.6 Dynamic mechanical analysis (DMA)

The DMA results provide three different important parameters: the storage modulus (*E*′), the loss modulus (*E*′′), and tan delta. These results provide a theoretical basis for the glass transition of composites.

**Fig. 15:**
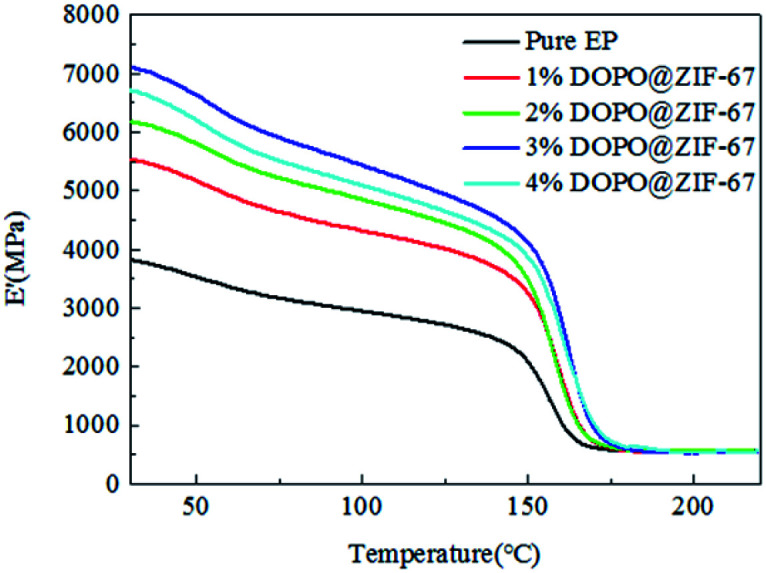
The storage modulus of flame-retardant EP composites.

The *E*′ determines the elastic or stiffness response of the material. As can be seen from Fig. 15, at the beginning, all the tested composites maintained their stiffness. However, the storage modulus has been falling. From about 150 °C, the resin began to soften, and the storage modulus dropped sharply. This behavior is caused by an increase in temperature, which leads to an increase in the molecular mobility of the material.^30,31^ As can be seen from the Fig. 15, the softening temperatures of the composites are very close. However, different composite formulas have different degrees of stiffness reduction. The results showed that before the softening point, the stiffness of the flame-retardant composites are all greater than that of the pure epoxy, but the stiffness of the flame-retardant composite is the highest with the addition of 3% of the flame retardant, indicating that the increase of the flame retardant content can improve the stiffness of the material, but there is a limit.

We speculate that the addition of flame retardant can effectively improve the stiffness of composite, but the improvement of stiffness is affected by two factors, one is the content of flame retardant, the other is the compatibility of flame retardant and composite. The more the content of flame retardant is, the more the stiffness will be improved, but at the same time, the more the flame retardant is, the interface compatibility between the flame retardant and the EP will be affected, so the stiffness will be reduced. When the content of the flame retardant is less than 3%, the influence of the content of the flame retardant is larger, so the stiffness of the composites is increasing.

**Fig. 16:**
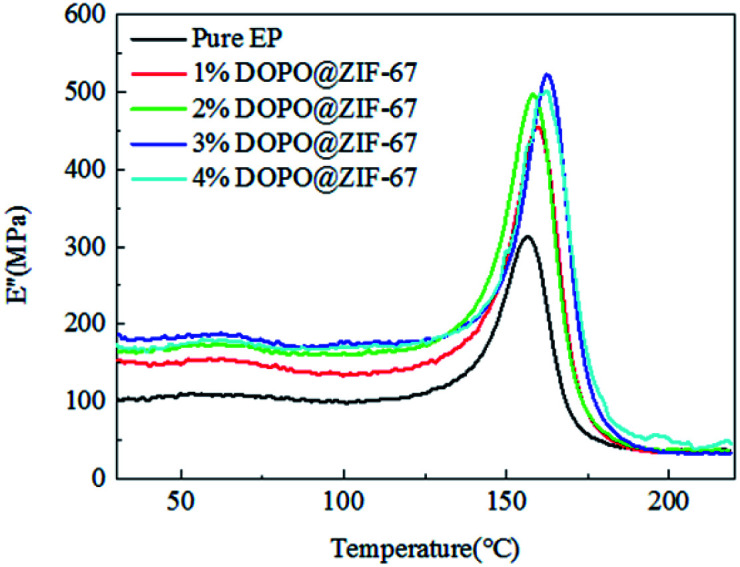
The loss modulus of flame-retardant EP composites.

The loss modulus is a measure of energy loss per cycle by stress deformation. It measures the loss of energy when a material deforms under heat. The loss modulus of all composites is shown in Fig. 16. Comparing the storage modulus with the loss modulus, it can be seen that as the storage modulus decreases, the loss modulus increases.

**Fig. 17:**
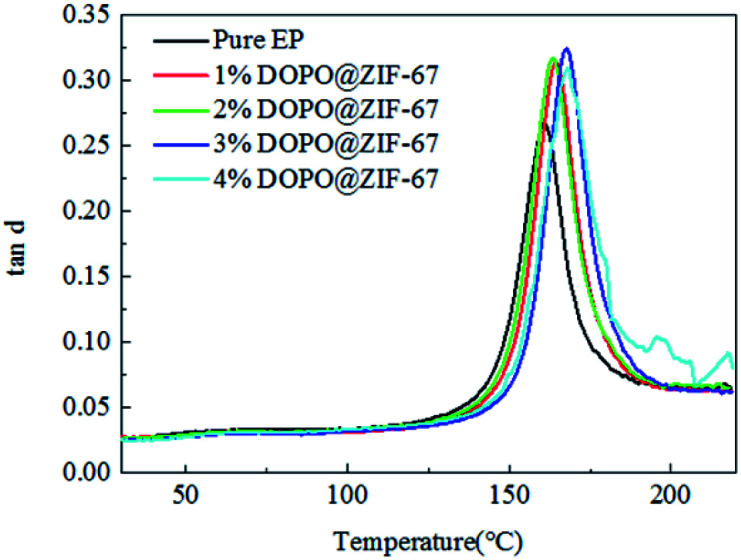
The tan delta of flame-retardant EP composites.

Fig. 17 shows the variation of tan delta with temperature for all composites. The tan delta measures the damping characteristics of the composites, and the ratio of the loss modulus to the storage modulus gives the tan delta. The damping coefficients of the flame retardant composites are higher than that of the pure EP, which proves that the flame retardant is well combined with the matrix material.^30,32–34^ At the same time, with the increase of flame retardant content, the damping coefficient of flame retardant composites shows a tendency of increasing first and then decreasing, which confirmed that the increase of flame retardant content enhanced the bond strength of flame retardant and matrix, but the increase is limited. The glass transition (*T*_g_) which is obtained from the peak of the tan delta curve shown in Table 6. After adding flame retardants, the *T*_g_ values were all increased, indicating that there is a good interfacial reaction between the DOPO@ZIF-67 and the matrix.

**Table 6:** The glass transition temperature of flame-retardant EP composites from the peak of tan delta

Sample name	Pure EP	1% DOPO@ZIF-67	2% DOPO@ZIF-67	3% DOPO@ZIF-67	4% DOPO@ZIF-67
*T*_g_ (°C)	160.8	164.5	163.4	167.7	168.2

### 3.7 Mechanical properties

**Fig. 18:**
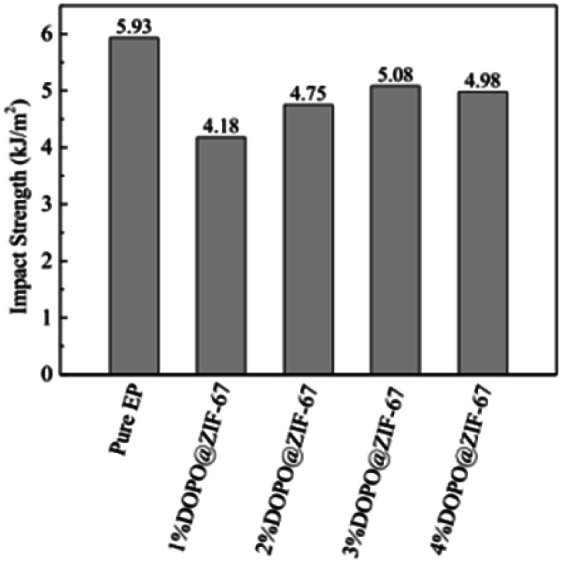
The impact strength of EP composites.

The mechanical properties of flame-retardant EP composites determine their application field.^35^ The influence of DOPO@ZIF-67 on the mechanical properties of EP was studied by simply supported beam impact testing machine. As the impact strength of epoxy thermosets shown in Fig. 18, the addition of DOPO@ZIF-67 has a slight adverse effect on the unnotched impact strength. Compared with pure EP, the impact strength of all the flame retardant modified EP composites have reduced. The impact strength of EP composites increases first and then almost remained stable with the increase of flame retardant content. C. Xu,^36^ R. Z. Huang,^37^ W. Chonkaew^38^*et al.*, all mentioned that the impact strength of composites decreased with the addition of fillers. The impact strength of 1% DOPO@ZIF-67/EP is lower than that of pure EP due to the presence of fillers. However, the impact strength is increased with the increase of the DOPO@ZIF-67 content, which is due to the fact that the presence of DOPO made the DOPO@ZIF-67 have better compatibility with the EP matrix. Therefore, the appropriate interfacial adhesion between DOPO@ZIF-67 and EP could effectively transfer the stress from the resin matrix to the flame retardant.^37^ Considering the high flame retardant dosage in the flame retardant composites, the impact performance of 4% DOPO@ZIF-67/EP to DOPO@ZIF-67/EP is not as expected. It can be explained that the high concentration of DOPO@ZIF-67 leads to discontinuity of the matrix and stress concentration in this region, resulting in the reduction of the impact strength of the EP composites.^21,36^ The impact strength values of EP composites are shown in Fig. 18.

## 4. Conclusions

The modification of EP was investigated by DOPO@ZIF-67 which formed by the reaction of DOPO and ZIF-67. The vibration absorption peak of the imidazole ring in the FTIR spectrogram, the representative reflections in XRD spectrum and the microstructure of the product in the SEM images confirmed the formation of ZIF-67. The formation of DOPO@ZIF-67 was confirmed by the vibration absorption peak of C–P and the microstructure of the product in the SEM images. The percentage of the chemical elements of DOPO@ZIF-67 obtained from XRF test showed that the covering rate of DOPO in DOPO@ZIF-67 is 40.57%. The addition of DOPO@ZIF-67 improved the flame retardant performance of EP, compared with the pure EP, 4% DOPO@ZIF-67/EP passed the UL94 V-0 level and the value of LOI reached 32.1%. In addition, DOPO@ZIF-67 can promote the carbonization process of flame retardant EP composites. As can be seen from the SEM pictures of char residue, with the addition of DOPO@ZIF-67, more continuous and dense microscopic structure is formed, which helps to form a barrier to insulate heat and enhancement of physical barrier effect. DMA test results showed that the addition of flame retardant DOPO@ZIF-67 can effectively improve the stiffness and damping coefficient of EP, but the addition of DOPO@ZIF-67 has a slight adverse effect on the unnotched impact strength of EP composites, which indicates that the rigidity of the EP flame retardant modified materials is improved. The nano porous skeleton structure of DOPO@ZIF-67 can endow EP with excellent stiffness and flame retardant properties, at the same time, the existence of DOPO makes ZIF-67 and EP have a better combination. In conclusion, we found that the DOPO@ZIF-67 promoted the carbonization process of the EP flame retardant composites, and the rigidity and damping coefficient of EP composites are also improved compared with pure EP, the improvement of these three properties makes the application scope of EP expand and provided a reference for the modification of EP.

## Conflicts of interest

The authors declare no conflict of interest.

## Supplementary Material
